# Impact of Nurses' Peak Workload and Time Pressure on Work Exhaustion and Turnover Intention

**DOI:** 10.1155/jonm/2311721

**Published:** 2025-09-08

**Authors:** Hao-Yuan Chang, Yi-An Lin, Wen-Pin Yu, Hsin-Hsu Wu, Gen-Yih Liao, Ann Rudman, Ching-I Teng

**Affiliations:** ^1^School of Nursing, National Taiwan University, Taipei, Taiwan; ^2^Department of Second Degree Bachelor of Science in Nursing, National Taiwan University, Taipei, Taiwan; ^3^Department of Nursing, National Taiwan University Hospital, Taipei, Taiwan; ^4^Department of Industrial and Business Management, Chang Gung University, Taoyuan, Taiwan; ^5^Department of Nursing, Chang Gung Medical Foundation, Chang Gung Memorial Hospital, Linkou, Taiwan; ^6^Chang Gung University of Science and Technology, Taoyuan, Taiwan; ^7^Teaching and Research, Department of Nursing, Chang Gung Memorial Hospital, Linkou, Taiwan; ^8^Department of Information Management, Chang Gung University, Taoyuan, Taiwan; ^9^Division of Pediatric Critical Care Medicine, Chang Gung Memorial Hospital, Linkou, Taiwan; ^10^Department of Clinical Neuroscience, Karolinska Institute, Stockholm, Sweden; ^11^Department of Caring Sciences, School of Health and Welfare, Dalarna University, Falun, Sweden; ^12^Graduate Institute of Management, Chang Gung University, Taoyuan, Taiwan; ^13^Department of Nursing, Chang Gung Memorial Hospital, Linkou, Taiwan; ^14^Department of Business and Management, Ming Chi University of Technology, Taishan, Taiwan

**Keywords:** nurse, peak workload, survey, time pressure, turnover intention, work exhaustion

## Abstract

**Background:** Nurse shortage has been a worldwide problem. Work exhaustion fuels nurses' turnover intentions. However, there is limited understanding of how peak workload and time pressure contribute to work exhaustion and turnover intentions.

**Aims:** To test the influence of peak workload and time pressure on work exhaustion and nurses' turnover intentions.

**Design:** A research design of using follow-up surveys was employed.

**Methods:** Complete survey responses were obtained from 423 nurses in Taiwan during 2023 and 2024. Structural equation modeling was used for analysis.

**Results:** Peak workload was related with time pressure. Both factors were positively associated with work exhaustion, which in turn was positively associated with turnover intentions. Interestingly, both factors were not directly related to turnover intentions.

**Conclusion:** Work exhaustion is pivotal in the impact of peak workload and time pressure on turnover intention. The unique value of this study is to identify the key role of work exhaustion in fueling turnover intention.

**Implications for Nursing Management:** The results inform the nurse managers that they could consider incorporating more flexibility into nursing routines to reduce peak workload. Additionally, increased support from auxiliary staff, recovery environment, and work flexibility may help alleviate nurses' perceived time pressure or workplace exhaustion.

## 1. Introduction

The global nursing workforce has experienced a significant shortage, projected to reach 4.5 million nurses by 2030 [1]. To alleviate this issue, it is crucial to reduce turnover intention (the inclination to leave employment at the hospital) among nurses.

The literature identifies several factors that contribute to nurses' turnover intention: quality of life threats [2], job stress [3], understaffing [4], and psychological distress [5]. Among these factors, exhaustion has been widely recognized [6, 7], highlighting the need to examine both work exhaustion (a feeling of being exhausted after work) and turnover intention. Using attention restoration theory (ART), we identified predictors of work exhaustion. Consistently high workloads, which impose *time pressure* (stress from having insufficient time to complete tasks), and high *peak workload* (burdens during peak hours, including assignments, personnel arrangements, work-rest schedules, and patient care) may affect work exhaustion. However, it is unclear whether time pressure and peak workload, individually or together, impact work exhaustion and turnover intention, thus revealing a knowledge gap. Addressing the gap informs nurse managers to design effective strategies to alleviate work exhaustion and nurses' turnover intention.

Hence, our research aims to test the influence of peak workload and time pressure on work exhaustion and nurses' turnover intention. Using complete responses from 423 nurses across two survey waves, this study identified work exhaustion as the key process variable in the relationship among peak workload, time pressure, and turnover intention. This finding informs nurse managers that addressing work exhaustion is pivotal when designing policies to lower turnover intention among nurses.

## 2. Research Background

Nurses' turnover is a critical issue, as the global nurse shortage has been a persistent problem [1]. Nurses' turnover intention has negative effects on care quality [8, 9] and is therefore important to nurse managers.

Nurses' turnover intentions are strongly linked to degraded workplace conditions, such as lack of support from employers [2] and work exhaustion [6, 7]. Among these factors, work exhaustion exerts strong influence on nurses' turnover [7], justifying its inclusion in our model.

Work exhaustion reflects the depletion of emotion and energy [6]. This depletion likely results from excessive workload [10, 11] and/or highly demanding workplaces [12]. Excessive workload places significant pressure on nurses due to insufficient time to complete tasks [13], justifying our inclusion of time pressure.

Previous studies indicate that time pressure is a key stressor in the workplaces [14]. Time pressure increases the likelihood of missed care and nurses' turnover intention [13]. These studies underscore the importance of addressing time pressure.

Work exhaustion manifests as increased tiredness and deteriorated mental health [11], often resulting from numerous tasks to be completed within a given timeframe [15], e.g., peak-time medication pass, which is used to gauge workload [16]. This specific condition of increased tasks and tiredness motivated us to include work exhaustion in this study.

Past studies have clarified the importance of the variables in our study. Specifically, turnover intention degraded care quality [8, 9]. Time pressure affects patient safety [13]. Work exhaustion dissatisfies nurses [17]. Peak workload likely increases work intensity, which degrades nurses' health [11]. These findings in the literature support the importance of all variables in our study.

While past studies have examined the impact of work exhaustion on turnover intention [6, 7], they have less frequently explored its antecedents: work intensity [11] and demanding workplaces [12]. This indicates a research gap whether work exhaustion originates from peak workload. Our study addresses this gap by examining the impact of peak workload on work exhaustion, advancing our understanding of novel strategies to reduce work exhaustion.

### 2.1. Attention Restoration Theory (ART)

ART posits that individuals need time to recover their directed attention to be used to process information from their environments, while directed attention can be most effectively restored in a natural environment [18]. The underlying mechanisms include a psychological distance from daily tasks [19] and “microbreak” activities at workplaces, e.g., relaxation and social activities and [20] showing the applicability of ART to contexts not involving natural environments.

Rapid depletion of directed attention reduces the capacity to process incoming information [21], potentially leading to mental issues such as depression [19]. ART has been applied in various contexts, including management [21] and nursing [19], indicating its relevance to our research on nursing management issues.

Workplaces constantly demand nurses' directed attention due to the overall workload, which causes *time pressure*, and the concentration of tasks during peak hours, i.e., *peak workload*. Hence, we focused on these two antecedents to examine how they contribute to work exhaustion and nurses' turnover intention.

Peak workload reflects a surge in workplace tasks typically occurring during peak hours [16], thus increasing work intensity [11] and thereby requiring increased working speed and consuming significant energy [12]. ART research indicates that unrestored attention capability decreases individuals' ability to process additional information [21], leading nurses to perceive increased time pressure. Thus,

  H1: Peak workload is positively related to time pressure

Nursing routines have specific schedules for patient care and therefore cannot be significantly delayed. Hence, peak workload prompts nurses to work continuously to meet the schedule, increasing work intensity and therefore imposing threat to nurses' health [11]. According to ART, continual working with reduced breaks hinders restoration of energy [18], causing a feeling of exhaustion due to work [22]. Moreover, peak workload implies work intensity, which deteriorates nurses' mental health [11], supporting the link between peak workload and work exhaustion. Thus,

  H2: Peak workload is positively related to work exhaustion.

Time pressure indicates a state of perceiving psychological stress due to the obligation to complete tasks in a short time [13]. Prolonged time pressure indicates that individuals continue losing their attention resources and have insufficient time to restore their directed attention. This is further supported by the literature, suggesting that time pressure may cause missed care [13]. In this case, ART posits that individuals will experience reduced energy for work [18], causing work exhaustion. Moreover, constant time pressure implies increased workload, which causes emotional exhaustion regarding the workplace [15]. Thus,

  H3: Time pressure is positively related to work exhaustion.

Peak workload requires extraordinary energy to meet a surge in workplace tasks, likely causing a whirlwind of overwhelming emotions and boosting turnover intention [23]. This large expenditure of energy goes against individuals' tendency to conserve personal resources [24], posing a threat to quality of life and fueling turnover [2]. Hence, peak workload likely degrades nurses' love for the job or affective commitment, thus fueling nurses' turnover intention [12]. Thus,

  H4: Peak workload is positively related to turnover intention.

Time pressure is a psychological stress due to limited time to finish many tasks [13]. Time pressure is frequently associated with understaffing, which is related to turnover intention [4]. Time pressure likely increases missed care [13]. To reduce the likelihood of missed care, nurses are motivated to use their personal time, e.g., reducing breaks. According to the conservation of resources perspective, using personal resources causes dissatisfaction [24], which seeds turnover intention [3]. Thus,

  H5: Time pressure has a positive association with turnover intention

Work exhaustion reflects the depletion of energy [22]. This depletion is contrary to individuals' tendencies [24], causing psychological distress, which is a key to increasing nurses' turnover intention [5]. Moreover, work exhaustion likely dissatisfies nurses [17]. Dissatisfaction should increase turnover intention. Thus,

  H6: Work exhaustion is positively associated with turnover intention

**Figure 1:**
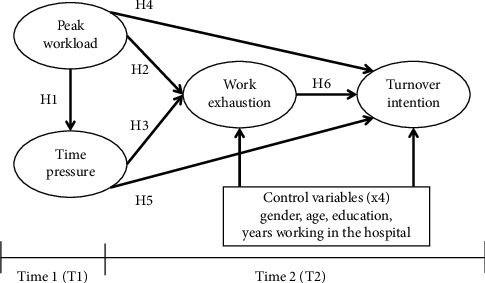
Research model.

Peak workload is new to the research literature. Hence, hypotheses pertaining to it are new (H1, H2, and H4). This study uniquely compares the total impact of time pressure and peak workload. Thus, the other paths in the model are also needed.  Figure 1 illustrates the research model. As control variables are used for ruling out alternative explanation, they are used in our model as explaining endogenous variables but not exogenous ones.

## 3. Methods

### 3.1. Research Site, Sampling, and Data

Our research utilized multiple waves of survey design. The first wave of data was collected in November, 2023, and the second wave in November, 2024. The research was conducted at a medical center in Taiwan.

Regarding research ethics, several measures were taken to ensure compliance. First, a government-certified institutional review board (IRB) has reviewed and approved this research (202202337B0C602). Second, prospective participants were briefed on the research aims and given the opportunity to consent to participation, completing the informed consent process. Third, research assistants were recruited from outside the hospital to avoid pressuring nurses to participate. Fourth, completed questionnaires were returned directly to the assistants, ensuring confidentiality.

The inclusion criteria were nurses who work full-time in the units we were permitted to approach. The exclusion criteria were based on their titles. We excluded nursing supervisors, nursing students, and nurse interns to ensure comparability among nurse participants.

This study employed a proportionate random sampling method to maintain the representativeness of the sample while keeping substantial randomness. Approximately, the same proportion of nurses was drawn from each unit to comprise the sample. Sampled nurses were approached, briefed on the research aims, and given the option to consent participation. No replacements were made when sampled nurses declined participation. We drew 600 nurses, and eventually, 448 nurses participated in both waves of the survey. Among them, we obtained 423 complete responses. The high response rate reflects the absence of nonresponse bias. The collected responses were used for the analyses. Each nurse who returned a completed questionnaire awarded a gift certificate valued at NT$200 as an expression of gratitude from the researchers.

The sample size was estimated using a university-affiliated website. Specifically, we used the website by Wein University (https://reurl.cc/G5MbGy) to calculate the required sample size for testing the correlation between exhaustion and turnover intention. The “true” correlation was set as 0.24 [25]. Alpha was set at 0.05, and testing power at 0.80. The required sample size was 135, which exceeded our sample size.

### 3.2. Measurement

All measurement items were rated on a five-point response scale. The anchors are as follows: (1) denoting “*strongly disagree,*” (2) denoting “*disagree,*” (3) denoting “*neutral,*” (4) denoting “*agree,*” and (5) denoting “*strongly agree.*” Scores for each construct were evaluated using the average. For each concept, a high score represents its high level. Control variables of our research are age, gender, education, and the number of years of service in the current hospital.  Table A1 carries the measurement items and loading from confirmatory factor analysis (CFA).

### 3.3. Psychometric Properties

This research calculated the loadings and cross-loadings for the measures using exploratory factor analysis (EFA) and maximum likelihood estimators. The loadings on the assumed factors were significantly larger than the cross-loadings. This pattern, as shown in Table 1, indicates a robust factor structure, which preliminarily supports the measurement quality.

The measurement items in our research were adapted from previous studies. The measurements had good reliability in the literature. Specifically, time pressure was assessed using four items from Huang et al. [13], which had Cronbach's alpha of 0.92 and composite reliability (CR) of 0.95, which is also called McDonald's omega [26]. Peak workload was assessed using eight items by Alomari et al. [27], with Cronbach's alpha of 0.93 and CR of 0.95. Turnover intention was evaluated using three items from Chang et al. [28] that had Cronbach's alpha of 0.87 and CR of 0.92. Work exhaustion was assessed using four items from Ahuja et al. [22] with Cronbach's alpha of 0.93 and CR of 0.95. All constructs in our study exhibited Cronbach's alpha values > 0.87 and CR > 0.92, indicating sufficient reliability.

We also tested measurement reliability. CFA was chosen to examine the validity and reliability of our model.  Table 2 summarizes how the research concepts correlate with each other. All constructs had Cronbach's alpha values > 0.87, average variance extracted (AVE) values > 0.69, and CR > 0.92, indicating sufficient reliability. Moreover, the square roots of the AVE exceeded the correlations, demonstrating confident discriminant reliability.  Table A1 shows that all items had indicator loadings greater than 0.76. Moreover, AVE values were all > 0.50, demonstrating adequate convergent validity. The measurement model showed adequate model fit, i.e., comparative fit index (CFI) = 0.96, non-normed fit index (NNFI) = 0.95, and standardized root mean squared residual (SRMR) = 0.041, fulfilling the criteria established in the literature [29].

Table 2 presents the analytical results of correlations and reliability tests. All correlations ranged from 0.16 to 0.68, indicating no common method bias (CMB). We tested CMB by employing Harman's single factor test which showed that all factors explained less than half of the data variance. The present study further tested CMB using the method by Podsakoff et al. [30], which involves using a common method factor to explain all measures and thereby building a rival model, but it fits the data significantly worse than our measurement model, with a chi-square difference of 4071.57, exceeding the threshold value of 12.59, for six degrees of freedom in the model difference. Both testing results support that CMB unlikely affected our measurement quality.

### 3.4. Data Analysis

The LISREL program v.8.80 (Scientific Software International, Skokie, IL, USA) was used to implement the structural equation modeling (SEM) method for testing the hypotheses. The endogenous variables were work exhaustion and turnover intention. We set peak workload and time pressure as the exogenous variables. A significance level of 0.05 was set for the analysis. Only complete responses were used, ensuring there were no missing values.

## 4. Results

### 4.1. Participants' Characteristics

Table 3 depicts the characteristics of the study participants. We collected 423 responses. Most participants were women (96.2%) and under 40 years old (71.9%), which aligns with the local nursing population. Among the local nurse population, 94% were women with an average age of 38.6 years [31]. A large proportion of female nurses is common, as seen in Korea, where 89.2% of nurses were female [32]. The majority of our participants had a university or college degree or higher (87.5%) and had worked in the hospital for less than 20 years (79.9%). The participants work in wards (*n* = 274), intensive care units (*n* = 113), operating rooms (*n* = 28), or special units, e.g., chemotherapy center, hyperbaric oxygen therapy center, delivery room, or burn care center (*n* = 8).

### 4.2. Hypothesis Testing

**Figure 2:**
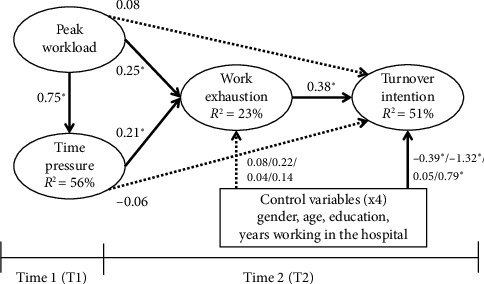
Testing results. Note: ^∗^*p* < 0.05. The dotted lines indicate paths carrying insignificant coefficients.

Table 4 and Figure 2 present the outcomes of the study hypotheses testing. The results explained marked variance of the endogenous concepts: 56% for time pressure, 23% for work exhaustion, and 51% for turnover intention. These percentages represent effect sizes that should be assessed as medium to large [33]. We found that variance inflation factors (VIFs) were all smaller than five, not revealing severe multicollinearity [34]. Moreover, the SEM method has included correlations among the exogenous variables, alleviating the issue of multicollinearity. Most hypotheses were supported. Peak workload was positively related to time pressure (ß = 0.75, *p* < 0.001) and work exhaustion (ß = 0.25, *p* < 0.001), supporting H1 and H2. Time pressure was positively related to work exhaustion (ß = 0.21, *p*=0.003), supporting H3. Peak workload was not related to turnover intention (ß = 0.08, *p*=0.11), not supporting H4. This may be because nurses perceive peak workload as temporary and resolvable. Time pressure did not have a relation with turnover intention (ß = −0.06, *p*=0.15), not supporting H5. This can have a reason that nurses develop resilience to cope with high time pressure. Work exhaustion had a positive relation with turnover intention (ß = 0.38, *p* < 0.001), supporting H6. It is acceptable that some hypotheses are not supported [32].

We used the bootstrapping method to test mediations. Our analysis revealed that work exhaustion was a significant mediator in the impact of peak workload and time pressure on turnover intention, supporting our model's inclusion of work exhaustion as a mediator.  Table A2 lists the mediation analytical results.

We used confidence intervals of the point estimates to compare the total effects of time pressure and peak workload on turnover intention. Our findings showed that peak workload had a significantly stronger total effect on turnover intention (0.11 > 0 .01, *p* < 0.05). Thus, we concluded that peak workload is more important than time pressure, showing its importance in our model.

## 5. Discussion

### 5.1. Findings and Major Contributions

Our research pioneers to examine the role of work exhaustion in the relationships between peak workload, time pressure, and turnover intention. We found that work exhaustion is pivotal, as it mediates the impacts of peak workload and time pressure on turnover intention, showing its pivotal role.

### 5.2. Theoretical Implications

Previous studies have found that nurses' turnover intention can be predicted by time pressure and/or excessive workloads [12, 13]. Comparatively, our findings consistently show that time pressure and workloads are important, but uniquely clarify that the underlying mechanism has increased work exhaustion.

Past studies on nursing management have clarified a number of factors affecting nurses' turnover intention: nurses' well-being [35] and workplace bullying [36]. These studies clarifies that social well-being is important for retaining nurses. Comparatively, our study is new in indicating that nurses' turnover intention may also be affected by work exhaustion, which originates from peak workload and time pressure.

The literature on time pressure indicated that the use of nurses' strength at workplaces may reduce time pressure [37]. Moreover, time pressure pushes individuals to work faster [13]. Our findings also examined the role of time pressure in the workplace, uniquely showing that the key process underlying the impact of time pressure is work exhaustion.

Research using ART found that breaks during intensive task completion help restore individuals' cognitive resources [18] and enhance performance [21]. Our results support these findings, providing evidence in a healthcare setting. Our study adds value to this theory by showing that lack of attention restoration can cause long-lasting negative outcomes, in addition to reduced positive outcomes.

### 5.3. Implications for Nurse Managers

The research was executed in a medical center, encompassing many specialized medical departments. Thus, the study results could be generalized to international contexts of large medical centers. Our findings provide actionable implications, as peak workload and time pressure can be managed.

We found that peak workload contributes to work exhaustion, thereby increasing nurses' turnover intention. Hence, nurse managers are advised to establish a mechanism for monitoring nurses' peak workload, for example, regularly obtaining nurses' feedback regarding peak workload. When excessive peak workload is detected, hospital management should be encouraged to devise means to reduce it, for instance, by offering more flexibility in scheduled care routines for nurses, which helps enhance quality of care [7].

The findings indicated that time pressure increases work exhaustion and subsequent turnover intention. Hence, nurse managers could introduce technologies that help share nurses' nonprofessional tasks, e.g., using robots to transport materials. Moreover, nursing assistants or other nonprofessional staff could be hired to assist nurses during peak time. Additionally, recovery environment and work flexibility may help alleviate nurses' perceived time pressure or workplace exhaustion.

The findings provide implications for multiple stakeholders. These implications also apply to hospital management who aim to retain nurses. Nurses could also remind themselves to take a break during busy care routines, e.g., taking a short walk in natural environments, as indicated by ART. Hospital management could increase nurses' access to a natural environment (e.g., a small garden). Doing so may effectively restore nurses' attentional resources, maintaining their passion for the profession and contributing more years working as nurses.

### 5.4. Limitations and Future Directions

We employed a two-wave follow-up research design, providing temporal separation that helps address common method issues. However, strong causality claims may need support from experimental designs, which future studies can utilize.

The study participants were representatives of a large medical center, ensuring coverage of as many units as possible. However, future studies can replicate our research in other countries to extend the external validity of our analytical results.

This study introduced and examined peak workload and its effects. Future research can further explore its predictors, providing more viable means to manage it.

Future studies using path analysis models should consider correcting for measurement error in the mediation and endogenous latent variables, either by including measurement error or obtaining multiple measures for use in latent variable modeling [38].

Our study did not aim to theorize or test serial mediations. Future studies may have this aim and further examine the possible serial mediations in our model.

## 6. Conclusions

Our study addresses the nurse shortage problem by examining the pivotal role of work exhaustion and proposing a novel means of reducing peak workload for managing nurses' turnover intention in practice. Overall, work exhaustion is the key to transforming workplace burdens (peak workload and time pressure) into turnover intention. Peak workload has a stronger impact than time pressure on turnover intention. Future studies should further explore workplace designs that may help alleviate peak workload.

## Figures and Tables

**Table 1:** Loadings and cross-loadings.

	1	2	3	4
Time pressure 1	**0.78**	0.33	0.02	0.17
Time pressure 2	**0.79**	0.35	0.01	0.15
Time pressure 3	**0.81**	0.35	0.06	0.08
Time pressure 4	**0.81**	0.30	0.07	0.15
Time pressure 5	**0.79**	0.34	0.05	0.13
Peak workload 1	0.25	**0.72**	0.04	0.06
Peak workload 2	0.28	**0.76**	0.05	0.07
Peak workload 3	0.43	**0.69**	0.11	0.11
Peak workload 4	0.25	**0.72**	0.05	0.17
Peak workload 5	0.20	**0.78**	0.03	0.23
Peak workload 6	0.25	**0.81**	0.07	0.09
Peak workload 7	0.23	**0.78**	0.07	0.20
Peak workload 8	0.26	**0.77**	0.01	0.15
Turnover intention 1	0.03	−0.01	**0.86**	0.13
Turnover intention 2	0.04	0.10	**0.91**	0.19
Turnover intention 3	0.07	0.13	**0.84**	0.21
Work exhaustion 1	0.15	0.16	0.13	**0.87**
Work exhaustion 2	0.15	0.17	0.13	**0.88**
Work exhaustion 3	0.11	0.17	0.18	**0.85**
Work exhaustion 4	0.14	0.17	0.20	**0.87**

*Note:* The bolded numbers are loadings on the theoretically assumed factors.

**Table 2:** Study concepts and their correlations.

	1	2	3	4
1. Time pressure	**0.88**			
2. Peak workload	0.68	**0.83**		
3. Turnover intention	0.16	0.20	**0.89**	
4. Work exhaustion	0.36	0.39	0.39	**0.91**

Mean	3.66	3.93	2.74	3.51
Standard deviation	0.90	0.81	1.15	0.97
Cronbach's α	0.92	0.93	0.87	0.93
Composite reliability	0.95	0.95	0.92	0.95
Average variance extracted	0.78	0.69	0.80	0.83

*Note:* The correlations are significant (*p* < 0.05). The average variance extracted (AVE) values are placed on the diagonal.

**Table 3:** Summary of the participant profile.

Characteristic	Classification	No.	%
Gender	Female	407	96.2
	Male	16	3.8

Age (years old)	≥ 20 and < 30	154	36.4
	≥ 30 and < 40	150	35.5
	≥ 40 and < 50	97	22.9
	≥ 50	22	5.2

Education	Junior college	53	12.5
	University or college	354	83.7
	Graduate institute or above	16	3.8

Years of service in the hospital	< 5 years	94	22.2
	≥ 5 and < 10 years	114	27.0
	≥ 10 and < 15 years	85	20.1
	≥ 15 and < 20 years	45	10.6
	≥ 20 years	85	20.1

*Note:* No. = number; % = percentage.

**Table 4:** Testing results of each hypothesis.

	Predictor	Criterion	Path	*p* value	Testing result
H1	Peak workload	Time pressure	0.75	< 0.001	Supported
H2	Peak workload	Work exhaustion	0.25	< 0.001	Supported
H3	Time pressure	Work exhaustion	0.21	0.003	Supported
H4	Peak workload	Turnover intention	0.08	0.11	Not supported
H5	Time pressure	Turnover intention	−0.06	0.15	Not supported
H6	Work exhaustion	Turnover intention	0.38	< 0.001	Supported

*Note:* Path = standardized path coefficient.

**Table 5:** Scales and items.

Construct	Item	*M*	SD	*λ*
Time pressure	I feel a lot of time pressure at work	3.8	1.02	0.87
	I feel like I am very busy at work	3.98	0.93	0.89
	I feel like it is not easy to complete the work at the set working hours	3.57	1.08	0.89
	I am always bustle at work	3.63	1.05	0.89
	I feel like there is not enough time to finish the work I am supposed to do	3.30	1.08	0.87

Peak workload	During peak hours, staffing and schedule arrangements are unpredictable	4.00	0.98	0.76
	During peak hours, I don't have enough time to provide emotional support to patients	4.09	0.99	0.84
	During peak hours, I don't have enough time to complete all my nursing tasks	3.63	1.05	0.84
	During peak hours, there is too much non-nursing work, i.e., paperwork	4.07	1.00	0.79
	During peak hours, there are not enough personnel to adequately complete the unit's requirements	3.94	1.04	0.84
	During peak hours, I don't have enough time to respond to the needs of patients' families	3.81	1.02	0.87
	During peak hours, I am constantly working and can't have time to rest	4.00	0.95	0.85
	During peak hour, I have to make decisions under pressure	3.94	0.94	0.85

Turnover intention	I plan to work in this hospital for a while^∗^	2.16	1.07	0.82
	I plan to work in this hospital for more than 5 years^∗^	2.84	1.35	1.00
	I plan to work in this hospital until retirement^∗^	3.23	1.41	0.85

Work exhaustion	I feel like I am mentally exhausted after work	3.67	1.03	0.92
	I feel mentally exhausted after daily work	3.75	1.01	0.93
	I feel exhausted about having to face a new day of work when I wake up	3.35	1.12	0.88
	I feel exhausted to work	3.27	1.10	0.91

*Note: M = *mean; *λ = *indicator loading.

Abbreviation: SD* = *standard deviation.

^∗^reversely coded.

**Table 6:** Mediation analytical results using bootstrapping methods.

Independent	Mediator	Dependent	LL	IE	UL	Result
Peak workload	Time pressure	Work exhaustion	0.04	0.15	0.26	Significant
Peak workload	Time pressure	Turnover intention	−0.08	0.04	0.16	Not significant
Peak workload	Work exhaustion	Turnover intention	0.13	0.20	0.28	Significant
Time pressure	Work exhaustion	Turnover intention	0.11	0.18	0.25	Significant

*Note:* LL denotes the lower limit of the 95% confidence interval of a mediation coefficient. The parameters in this table are not standardized.

Abbreviations: IE, indirect effect; UL, upper limit.

## Data Availability

The data that support the findings of this study are available from the corresponding authors upon reasonable request and ethical approval. The data are not publicly available due to privacy or ethical restrictions.

## References

[1] WHO (2024). Nursing and Midwifery. https://reurl.cc/26evzO.

[2] Bottega M., De Faveri A. P., Simeoni M., Danielis M. (2025). Why Nurses Discontinue Practice in Hospitals? Insights From a Qualitative Study. *International Nursing Review*.

[3] Bingöl Ü., Bilgin N., Çetinkaya A., Kutlu A. (2025). Variables that Predict Nurses’ Job Stress and Intention to Leave During the COVID-19 Pandemic in Turkey. *Journal of Advanced Nursing*.

[4] Pindek S., Hayman M. R., Howard D. J., Arvan M. L., Spector P. E. (2025). Understaffing as a Two-Dimensional Phenomenon: A Cross-Sectional Study of Hospital Nurses’ Manpower and Expertise Understaffing. *Journal of Advanced Nursing*.

[5] Edwin H. S., Trinkoff A. M., Mills M. E., Zhu S. (2025). Psychological Distress Symptoms in Nurses and Their Intention to Leave: A -Sectional Secondary Data Analysis. *Journal of Advanced Nursing*.

[6] Jiang D., Kira J. (2024). New Nurse Turnover Intention and Related Factors in Japan and China: Focusing on Nursing Practice Environment and Burnout. *Journal of Nursing Research*.

[7] Rodríguez-Fernández M., Herrera J., de las Heras-Rosas C., Ciruela-Lorenzo A. M. (2024). Practical Implications of the Organizational Commitment Model in Healthcare: the Case of Nurses. *Journal of Nursing Management*.

[8] Bae S.-H. (2025). Association Between Nurse Turnover and Nurses’ Perception of Patient Outcomes in Acute Care Hospitals in South Korea: A Cross-Sectional Study. *Journal of Nursing Care Quality*.

[9] Huang T.-L., Wong M.-K., Shyu Y.-I. L., Ho L.-H., Yeh J.-R., Teng C.-I. (2021). Reducing Turnover Intention to Improve Care Outcome: A -Wave Study. *Journal of Advanced Nursing*.

[10] Khan Y., Bruyneel A., Smith P. (2022). Determinants of the Risk of Burnout Among Nurses During the First Wave of the COVID-19 Pandemic in Belgium: A -Sectional Study. *Journal of Nursing Management*.

[11] Yun M., Kim W., Yu B., Choi E.-H. (2024). A Delphi Study on the Changes in Work, Organizational Culture, and Health Issues of Nurses at Tertiary Hospitals in South Korea During the COVID-19 Pandemic. *Journal of Nursing Management*.

[12] Bruyneel A., Dauvergne J. E., Bouckaert N. (2025). Association of Burnout and --Leave the Job With Objective Nursing Workload and Nursing Working Environment: A -Sectional Study Among Intensive Care Nurses. *Journal of Clinical Nursing*.

[13] Huang T.-L., Wu C.-N., Lee I.-C. (2022). How Robots Impact Nurses’ Time Pressure and Turnover Intention: A Two-Wave Study. *Journal of Nursing Management*.

[14] -Vidal M., -Muñoz M., Armas-Moreno C. (2025). Practices Related to Bladder Catheterisation Among Swedish Health Professionals: A Questionnaire Survey. *International Journal of Urological Nursing*.

[15] Molala W., Downing C. (2024). Lived Experiences of Intensive Care Professional Nurses Caring for COVID-19 Patients in Private Hospitals in Gauteng, South Africa: A Phenomenological Study. *Journal of Nursing Management*.

[16] Eck C. S., Knox M. K., Mehta P. D., Petersen L. A. (2024). Estimating the Relationship Between Nurse Staffing and Medication Pass Workload Using National Barcode Data. *Nursing Research*.

[17] Aloisio L. D., Coughlin M., Squires J. E. (2021). Individual and Organizational Factors of Nurses’ Job Satisfaction in Long-Term Care: A Systematic Review. *International Journal of Nursing Studies*.

[18] Kaplan S. (1995). The Restorative Benefits of Nature: Toward an Integrative Framework. *Journal of Environmental Psychology*.

[19] Gonzalez M. T., Hartig T., Patil G. G., Martinsen E. W., Kirkevold M. (2010). Therapeutic Horticulture in Clinical Depression: A Prospective Study of Active Components. *Journal of Advanced Nursing*.

[20] Kim S., Park Y., Niu Q. (2017). Micro-Break Activities at Work to Recover From Daily Work Demands. *Journal of Organizational Behavior*.

[21] Tang P. M., Klotz A., McClean S., Lee R. (2024). From Natural to Novel: the Cognition-Broadening Effects of Contact With Nature at Work on Creativity. *Journal of Management*.

[22] Ahuja M. K., Chudoba K. M., Kacmar C. J., McKnight D. H., George J. F. (2007). IT Road Warriors: Balancing Work-Family Conflict, Job Autonomy, and Work Overload to Mitigate Turnover Intentions. *MIS Quarterly*.

[23] Lim S. H., Zainal H., Lee L. J. (2025). Second Victim Experiences and Impact Among Acute Care Nurses: An Exploratory Study. *International Nursing Review*.

[24] Hobfoll S. E. (2001). -Self in the Stress Process: Advancing Conservation of Resources Theory. *Applied Psychology*.

[25] Chang H.-Y., Shyu Y.-I. L., Wong M.-K., Friesner D., Chu T.-L., Teng C.-I. (2020). Influence of Headaches on Nurse Intentions to Leave the Profession and the Hospital: A cross-sectional Survey. *Contemporary Nurse*.

[26] Hayes A. F., Coutts J. J. (2020). Use Omega Rather than Cronbach’s Alpha for Estimating Reliability. But…. *Communication Methods and Measures*.

[27] Alomari A. H., Collison J., Hunt L., Wilson N. J. (2021). Stressors for Emergency Department Nurses: Insights From a -Sectional Survey. *Journal of Clinical Nursing*.

[28] Chang H.-Y., Lee I.-C., Tai S.-I. (2023). Professional Engagement: Connecting -Efficacy to Actual Turnover Among Hospital Nurses. *Journal of Advanced Nursing*.

[29] Bagozzi R. P. (2010). Structural Equation Models Are Modelling Tools With Many Ambiguities: Comments Acknowledging the Need for Caution and Humility in Their Use. *Journal of Consumer Psychology*.

[30] Podsakoff P. M., MacKenzie S. B., Lee J.-Y., Podsakoff N. P. (2003). Common Method Biases in Behavioral Research: A Critical Review of the Literature and Recommended Remedies. *Journal of Applied Psychology*.

[31] Taiwan Union of Nurses Association (Tuna) (2023). Gender and Age Statistics of Medical Personnel. https://reurl.cc/jQjQx2.

[32] Kim S., Jeong S., Jeong S. H. (2025). Changes in Nursing Practice Among Clinical Nurses After Experiencing a Patient Safety Incident: Partial Least Squares Structural Equation Modeling. *Journal of Nursing Management*.

[33] Cohen J. (1992). A Power Primer. *Psychological Bulletin*.

[34] Kyriazos T., Poga M. (2023). Dealing With Multicollinearity in Factor Analysis: The Problem, Detections, and Solutions. *Open Journal of Statistics*.

[35] Liu D., Zou M., Ma Y. (2025). The Mediating Role of Psychological Capital and Work Engagement in the Relationship Between -Being and Turnover Intention Among Nurses in China. *Journal of Nursing Management*.

[36] Dong S., Shen X., Zhao T., Zeng R., Chen M. (2025). Validating a Multidimensional Perspective of the Relationship Between Workplace Bullying, Professional Quality of Life, and Turnover Intention of Chinese Novice Nurses. *Journal of Nursing Management*.

[37] Chang H.-Y., Lee I.-C., Tai S.-I. (2025). Empowerment and Optimum Use of Strengths Reduce Nurses’ Time Pressure. *Journal of Advanced Nursing*.

[38] Cole D. A., Preacher K. J. (2014). Manifest Variable Path Analysis: Potentially Serious and Misleading Consequences due to Uncorrected Measurement Error. *Psychological Methods*.

